# Soil management modulates vineyard airborne fungal communities and impacts fungal disease pressure

**DOI:** 10.3389/fpls.2026.1770877

**Published:** 2026-04-10

**Authors:** Florencia Asinari, Gabriele Bellotti, Giorgia Fedele, Edoardo Puglisi, Tito Caffi

**Affiliations:** 1 Department of Sustainable Crop Production, Università Cattolica del Sacro Cuore, Piacenza, Italy; 2 Instituto Nacional de Tecnología Agropecuaria, Instituto de Patología Vegetal, Consejo Nacional de Investigaciones Científicas y Técnicas, Unidad de Fitopatología y Modelización Agrícola, Córdoba, Argentina; 3 Department for Sustainable Food Process, Università Cattolica del Sacro Cuore, Piacenza, Italy; 4 Research Center on Plant Health Modelling, Università Cattolica dal Sacro Cuore, Piacenza, Italy

**Keywords:** sustainable viticulture, compost, cover crops, soil tillage, microbial ecology, downy mildew, powdery mildew, black rot

## Abstract

Viticulture increasingly faces challenges posed by global warming and its effects on yield and berry composition, highlighting the need for sustainable yet effective management strategies. In a two-year field trial conducted in one vineyard, we compared conventional tillage (T) and no-till (NT) regimes with three inter-row treatments: compost mulch (C), pruning-wood mulch (PW), and living cover crops (CC), while using a high-efficiency captaspore vacuum sampler to track shifts in the airborne fungal diversity followed by visual assessment of fungal disease symptoms during each growing season. Under relatively dry seasonal conditions, compost mulching was associated with lower downy and powdery mildew pressure, whereas these differences were not detectable under wetter conditions. The use of cover crops appeared to modulate grape sugar content, resulting in lower bunch weight and higher °Brix. Tillage regime was associated with shifts in airborne fungal richness and composition, including changes in the relative abundance of taxa related to *Erysiphe necator*. However, seasonal climatic conditions explained a substantial proportion of the observed variability. Overall, soil tillage emerged as the dominant driver shaping the airborne fungal community composition, while compost mulching exerted disease-suppressive effects only under moderate climatic pressure. These findings suggest that soil management may modulate the soil–air microbial interface and disease pressure in a climate-dependent manner, rather than acting as a dominant standalone driver.

## 1 Introduction

In 2023, the global vineyard surface area has reached 7.2 million hectares, with Spain, France, and Italy having the most extensive vineyard coverage in Europe (OIV, 2024). Italy, the world’s second-largest wine producer, lately faces a notable decline in grape production, largely due to extreme weather events. Indeed, heavy rainfalls triggered severe downy mildew (DM) outbreaks in central and southern regions, while flooding and hail damaged Northen Italy productions (OIV, 2024). In addition to climate-related stressors, grapevine pathogens continue to exert substantial pressure on viticulture, as vineyards are still among the most pesticide-dependent cropping systems in Europe (Nefti et al., 2024). Efforts to maintain grape production while reducing chemical inputs therefore do not reflect a reduced phytopathological risk, but rather the need to limit the environmental footprint of disease management strategies and to align viticultural practices with sustainability and regulatory objectives (Cataldo et al., 2021).

In this context, Integrated Pest Management (IPM) strategies have gained significant attention. These approaches emphasize minimal and targeted pesticide use, favoring environmentally friendly alternatives with lower environmental impact and health-associated risks (Wan et al., 2025). Among these strategies, soil management practices, such as inter-row cover cropping and mulching have become widely adopted to suppress weeds, reduce tillage, and improve soil health and biodiversity (Liebhard et al., 2024). However, their outcomes are highly site-specific. A comprehensive meta-analysis found that inter-row vegetation can boost vineyard biodiversity and prevent soil erosion in some cases, but in others competition with vines for water and nutrients was observed, leading to mixed effect on yield (Winter et al., 2018). Similarly, the impact of cover crops on yield depends strongly on climate, species selection, and management practices (Winter et al., 2018).

Moreover, IPM practices such as cover crops and mulching provide more consistent, positive results in relation to pest and disease control. Cabrera-Pérez et al. (2022) demonstrated that cover crops can outperform tillage and herbicide-based approaches in weed suppression when persistence and ground cover are high. The use of mixed cover crop has resulted in reduced damage from *Lobesia botrana*, and *Empoasca vitis*, while supporting the presence of beneficial flying insects in vineyards (Ranca et al., 2022). Fungal disease management can also benefit from these IPM practices. Cover crops may act as physical barriers, reducing the primary inoculum dispersal, for example limiting splash-dispersal of *Plasmopara viticola* oospores into canopy (Rossi and Caffi, 2012), thereby reducing the number of chemical applications required throughout the season for plant protection (Furiosi et al., 2022). Another observed effect is the reduction of vine vigor that promotes canopy openness and air circulation, lowering incidence of both powdery mildew (PM) (*Erysiphe necator*) and grey mold (*Botrytis cinerea*) (Valdés-Gómez et al., 2008). In Mediterranean vineyards, conventional tillage has been associated with higher DM and PM infection rates compared to cover crop management and reduced tillage (Oliveira et al., 2021).

Beyond disease control, soil management practices can reshape microbial communities, and there is a growing interest in how these belowground practices can influence vineyards’ health. The introduction of cover crops has been often associated with greater soil microbial diversity (Liebhard et al., 2024), supporting beneficial taxa that compete with pathogens through niche exclusion or antifungal compounds production. Soil microbiota composition is tightly linked to management practices (Del Duca et al., 2024; Li, 2023; Veresoglou et al., 2015), and because microbes affect vine nutrition, metabolism, and even berry chemistry, they are now considered part of vineyard’s “terroir” (Alcalá-Jiménez et al., 2025; Van Leeuwen and Seguin, 2006; Ganugi et al., 2023). Maintaining vineyards’ microbiota is therefore not only ecologically important but also quality-related (Hendgen et al., 2018). However, while soil and rhizosphere microbiomes have been widely studied in association to IPM (Carbone et al., 2021; Rivas et al., 2022; BernasChina et al., 2023), little is known about how soil management practices influence the composition of the airborne microbiome between vineyard rows.

Soil serves as a vast reservoir of fungal propagules, and management-driven changes in soil moisture, organic matter, and microclimate can alter both the quantity and composition of spores available for aerial dispersal (Van Agtmaal et al., 2017). Cover crops and compost modify soil structure and humidity at the row base, thereby influencing spore release and persistence within the canopy (Novara et al., 2020). Since this aerial compartment represents the interface where spores spread, pathogens colonize new hosts, and microbial interactions occur, understanding it is critical for disease management.

Despite the growing body of literature on vineyard soil and rhizosphere microbiomes, the aerial fungal compartment remains largely overlooked, particularly in relation to soil management practices (Abdelfattah et al., 2019). Yet, this airborne interface represents a critical epidemiological layer, where soil-derived propagules, canopy-associated microbes, and plant pathogens converge and interact.

To address this knowledge gap, we carried out a two-season case study in a small experimental vineyard, comparing conventional management with either grass or compost cover across two soil tillage regimes. Our objective was to evaluate how soil management modulates disease pressure and influences airborne fungal community structure. To our knowledge, this is the first study to systematically assess the influence of inter-row management and tillage regimes on the composition of vineyard aerial microbial communities. While previous research has documented the effects of soil management on belowground microbiota and the potential of cover crops to reduce pathogen dispersal from soil surfaces, the characterization of aerial microbiota in response to these practices has largely been overlooked. Our findings extend the known influence of belowground interventions into the aerial microbial landscape, revealing how soil management transforms ecosystem processes and affects pathogen spread and ultimately grape quality.

## 2 Material and methods

### 2.1 Experimental design and weather data collection

The study was conducted for two consecutive growing seasons (2022-2023) in an experimental vineyard located at the campus of Università Cattolica del Sacro Cuore in Piacenza, Italy (Emilia-Romagna region, 45° 02’ 06.0” N, 9° 43’ 46.5” E). The vineyard, planted with the Merlot cultivar in 2012, was trained using the Guyot system, with a planting density of 1.2 m within rows and 2 m between rows.

The vineyard was divided into two soil tillage regimes: with tillage (T) and without tillage (NT). Within each regime, three different inter-row treatments were applied: i) pruning wood (PW) crumbled and redistributed along the inter-rows (considered the conventional management); ii) temporary cover crops (CC); and iii) compost (C) application derived from in-farm residues (**Table 1**). These Inter-row treatments were also carried out in the under-vines zone. The cover crop species selected were *Vicia sativa* L. and *Sinapis alba*, previously reported as effective in the control of fungal diseases of vineyards (Hasanaliyeva et al., 2024). Each treatment was applied in the inter-row of 11 plants, with a total of 33 plants for each regime.

**Table 1:** Description of inter-row treatments and applications of plant protection products.

Inter-row treatment	Description	Number of phytosanitary treatments per year	Product
Conventional: Pruning wood (PW)	Shredding of pruning residues and redistributed in the inter-row of plot PW on 10 March (2022) and on 3 April (2023).	2022: 2	**A**
		2023: 8	**B; C**
Innovative: Temporary Cover Crops (CC)	Spring mix of Vicia sativa L. (84%) and Sinapis alba (16%) at a density of 90 and 8 kg/ha, respectively. Sowing was on 28 March (2022) and 31 March (2023).	2022: 2	**A**
		2023: 7	**B; C**
Innovative: Compost (C)	Application of compost made at the end of 2021 with senescent and dry leaves from horticultural plants and vines, grass and vine pruning remain. The compost was applied on 10 March (2022) and 3 April (2023).	2022: 2	**A**
		2023: 7	**B; C**

A, Active ingredient Meptyldinocap - Commercial product Karathane Star, produced by Corteva Agriscience Italia S.r.l (concentration: 35,71% p/p; Label dose: 0,5- 0,6 l/ha).

B, Active ingredient Pure sulfur – Commercial product Tiogold disperss, produced by UPL Italia S.r.l. (concentration: 80 g/100g; Label dose: 4 Kg/ha).

C, Active ingredient Copper metal- Commercial product Airone, produced by GOWAN Italia S.r.l. (concentration from tetraramic oxychloride: 10 g/100g; Label dose: 1.5 Kg/ha).

Powdery Mildew (PM) management differed between years and vineyard management systems (**Table 1**). In 2022, PM was controlled with two applications of Meptyldinocap (Karathane Star, Corteva Agriscience Italia S.r.l; July 1 and July 5). In 2023, copper- and sulfur-based products were applied (Airone, Gowan Italia S.r.l and Tiogold disperss, UPL Italia S.r.l, respectively) with fungicide scheduling differing between innovative (CC and C) and conventional (PW) systems. For innovative treatments (CC and C), fungicide applications followed disease-risk forecasts from vite.net^®^ (Horta s.r.l.; Rossi et al., 2012); while conventional treatment (PW) followed the regional phytosanitary bulletin schedule. Between April and July 2023, PW vines were treated 8 times, while CC and C vines were treated 7 (starting on May 12).

Daily data of mean temperature, relative humidity (RH), wetness duration, rainfall, wind direction, and wind speed were recorded by an automated weather station (iMeteos; Pessl Instruments GmbH) located <1 km from the experimental system. Growth Stage (GS) of each vine was assessed weekly in the vineyards according to the scale of Lorenz et al. (1995), from inflorescence emergence (GS55) to berry ripening (GS83).

### 2.2 Disease assessment

Powdery mildew (PM), downy mildew (DM), and black rot (BR) were visually assessed weekly from bud break to harvest in both years across all treatments and tillage regimes. Disease incidence was calculated as the number of leaf or bunches with disease symptoms on the total of leaf or bunches evaluated. The severity was calculated using the EPPO standard diagrams (EPPO, 2000). Disease incidence and severity were used for calculating the Area Under the Disease Progress Curve (AUDPC) (Madden et al., 2007).

### 2.3 Harvest data

At harvest, bunch number and weight per vine were recorded for each treatment and regime. In 2023, sugar concentration (Brix°) was also measured through a manual refractometer (RX-5000, ATAGO U.S.A., Inc.) on three replicates of 30 berries per plant.

### 2.4 Airborne microbiome sampling

Airborne fungal spores were collected during the growing season of 2022 (May 26, June 21, and July 26) and 2023 (May 26, June 28, and July 24) using a handler air spore sampler (MICROFLOW 60, Aquaria s.r.l., Italy). Sampling dates were selected to coincide with key grapevine phenological stages, as well as periods of higher disease pressure, in order to capture airborne microbial communities most relevant to disease dynamics in the vineyard. The handler air spore sampler was positioned on a photographic tripod (450G, Slik, Japan) at 50 cm above the soil. This height was chosen with the aim of sampling the microbiome present between the soil management treatments and the vineyard canopy.

Air was aspirated at 30L/min for 10 minutes in each inter-row treatment and tillage regime, with the sampler placed 60 cm from vines. The sampler allowed the airflow to convey onto the surface of a 6 cm diameter plate containing Potato Dextrose Agar (PDA) medium, used solely as adhesive surface. Plates were immediately cooled in insulated container with ice packs and frozen at -20 °C upon laboratory arrival to prevent selective growth of fast-growing fungal taxa. Three replicates per inter-row treatments in each soil tillage regime were sampled, spaced approximately four meters apart. This resulted in 18 plates per sampling date (54 plates/year) for a total of 108 samples. Blank PDA plates were exposed only to laboratory air as negative controls and yielded no fungal growth (data not shown).

### 2.5 DNA amplification and Illumina high-throughput sequencing

To assess fungal community composition, molecular analysis targeting the Internal Transcribed Spacer 1 (ITS1) region of fungal ribosomal RNA (rRNA) was performed.

The total DNA of the 108 aerial samples was extracted using the FastDNA™ SPIN Kit for Soil (MP Biomedicals, Santa Ana CA, USA) according to the manufacturer’s protocol from 700 mg of PDA medium scraped from the plate’s surface. Isolated DNA in each sample was quantified using the Quant-iT™ HS ds-DNA assay kit (Invitrogen, Waltham, Massachusetts, USA) with a QuBit**™** fluorometer.

The extracted DNA was amplified via PCR, to assess the relative abundance of the fungal population of each sample, with the following set of primers: ITS-1 (5’-TCCGTAGGTGAACCTGCGG-3’) and ITS-2 (5’-GCTGCGTTCTTCATCGATGC-3’). The reaction mix had the following composition: 12.5 μl of Phusion Flash High-Fidelity Master Mix (Thermo Fisher Scientific, Inc., Waltham, MA, United States), 1.25 μl of each primer at the concentration of 10 μM, 1 ng of DNA template, and 7 μl of nuclease free water. The thermocycler was set as follows: initial denaturation at 94 °C for 4 min, followed by 35 cycles of denaturation at 94 °C for 30 s, annealing at 56 °C for 30 s, extension at 72 °C for 1 min; and a final extension at 72 °C for 7 min. The presence/absence of fungal DNA was verified on 1% agarose gel.

The High Throughput Sequencing (HTS) method comprised the use of indexed forward primer, modified by adding a unique 9 nucleic acid–base extension at the 5′ end, acting as a unique barcode. Tag primers enabled many samples to be sequenced in parallel without losing their origin once pooled. To reduce the possibility of anomalous PCR products, due to unspecific primers annealing caused by the 9 nucleic acid extension itself, a two-step PCR was performed as described in Berry et al. (2011), consisting of 28 PCR cycles with non-barcoded primers followed by 8 additional cycles with barcoded primers. The final PCR products were quantified with QuBit™ fluorometer and pooled in equimolar concentrations (30 ng per sample). The pooled amplicons were then purified using the solid phase reversible immobilization (SPRI) method with the Agencourt AMPure XP kit (Beckman Coulter, Italy) according to the manufacturer’s protocol. Amplicon libraries were prepared and sequenced by Novogene (Cambridge, UK), which added Illumina-compatible adapters and performed sequencing on the NovaSeq 6000 platform (Illumina Inc., San Diego, CA) generating 250 bp paired end reads.

### 2.6 Sequences processing

Barcode demultiplexing and base calling were performed with the MiSeq Control Software v2.3.0.3, RTA v1.18.42.0 and CASAVA v1.8.2 (Bartram et al., 2011). Raw pair-end sequences were merged with ‘pandaseq’ (Masella et al., 2012) with a minimum overlap of 30 bp and allowing a maximum of two mismatches. Sequence quality filtering, denoising, and amplicon sequence variant (ASV) calling were performed using DADA2 within QIIME 2 (Bolyen et al., 2019) with defaults parameters. Taxonomic assignment of ASVs was performed using the QIIME 2 feature-classifier plugin with pre-trained UNITE v10.0 reference database for fungi (https://unite.ut.ee/), applying a confidence threshold of 0.7 at all taxonomic levels. The resulting taxonomy table was used for downstream visualization and analyses on MicrobiomeAnalyst (Dhariwal et al., 2017). To account for differences in sequencing depth and remove low-abundance or invariant features, rarefaction was applied after ASV generation by subsampling all samples to the library size of the smallest sample (5,525 reads), following recommendations for marker-gene datasets with heterogeneous library size (Weiss et al., 2017). Rarefied data were used for α- and β-diversity analyses, while differential abundance analyses were performed on filtered count data using the normalization methods implemented within MicrobiomeAnalyst (e.g., DESeq2, metagenomeSeq). The dataset included discrete experimental factors with biological replicates, and no continuous variables.

### 2.7 Statistics and bioinformatic analyses

#### 2.7.1 Disease assessment and harvest data

The final values of AUDPC calculated for both incidence and severity data for each disease were subjected to factorial analysis of variance (ANOVA). AUDPC for leaves and bunches was summed to estimate the overall effect of tillage and treatments on plants. Years, tillage regime, and inter-row treatment were included as factors, considering the interaction between them. For the harvest data, an ANOVA was performed comparing the number, weight, and °Brix of each inter-row treatment within each soil tillage regime. Statistical analysis was performed using the statistical software RStudio (Rs. Team, 2020) an integrated development environment (IDE) for R (version 4.2.1 of R Core Team, 2021). *Post-hoc* comparisons were performed using Tukey’s HSD.

#### 2.7.2 Microbiome data

Microbiome data were pre-processed prior to statistical analysis to exclude low-quality or uninformative features. Specifically, ASVs were filtered out if they (i) had fewer than four reads in at least 20% of the samples, or (ii) exhibited less than 10% inter-quantile range across samples. These criteria were applied to remove ASVs likely derived from sequencing noise, low-level contamination, or features that were nearly invariant across experimental conditions. Airborne fungal diversity was assessed by calculating α-diversity using Shannon, Chao1, and Observed indices, with statistical significance evaluated with Welch’s ANOVA. β-diversity was computed using Bray-Curtis dissimilarity calculated on rarefied ASV counts converted to relative abundances for each sample, and the resulting distance matrix was visualized using Principal Coordinates Analysis (PCoA). Statistical differences in community composition were tested using pairwise PERMANOVA with 999 permutations. To identify taxa driving compositional shifts, LEfSe (Linear Discriminant Analysis Effect Size) was applied to rarefied relative abundances, with taxa considered significantly enriched if LDA scores exceeded 2.0 and p < 0.05 (Segata et al., 2011). For selected comparisons, LEfSe outputs were presented alongside β-diversity plots to highlight both global community shifts and the specific taxa driving them. The Metastats algorithm (White et al., 2009) was also applied to detect genera differing significantly across treatments, focusing on months with the highest disease pressure as indicated by AUDPC values. These analyses were conducted following microbiome analysis best practices in R as described by Wen et al. (2023).

## 3 Results

### 3.1 Weather conditions

**Figure 1:**
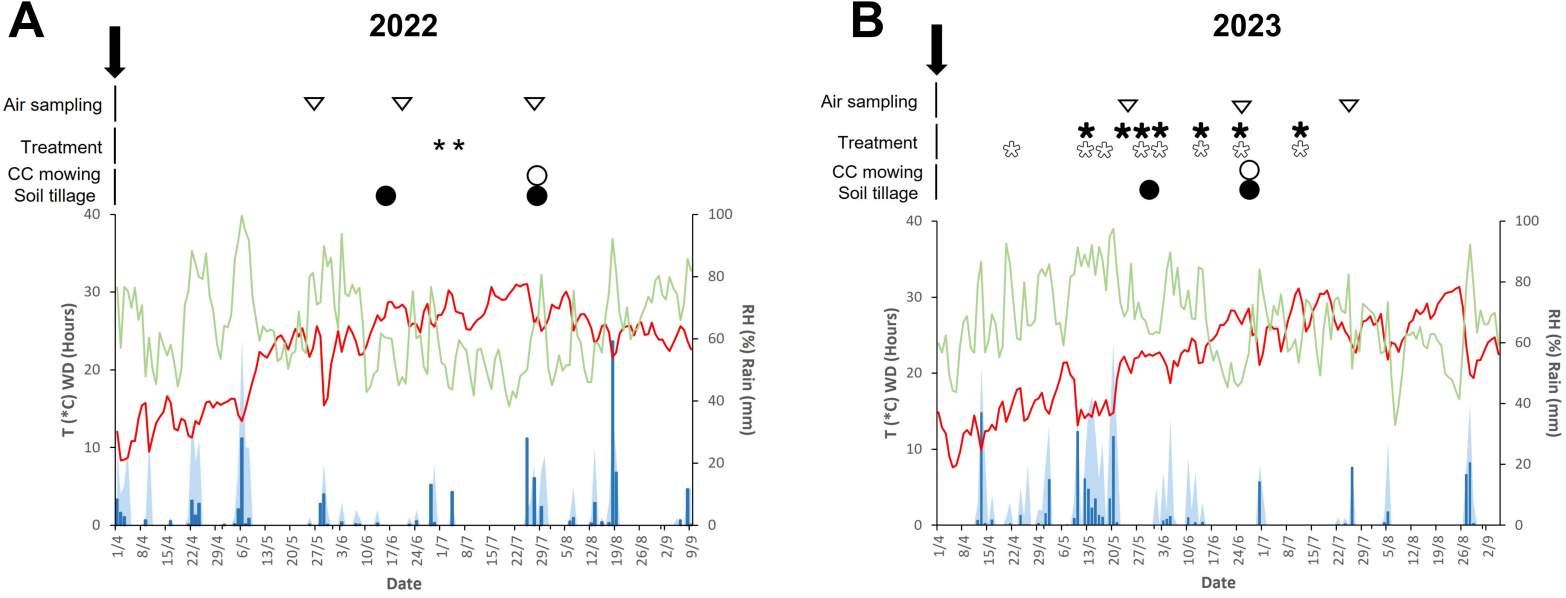
Activities related to inter-row treatments and weather data registered during the 2022 **(A)** and 2023 **(B)** growing seasons. The black arrow indicates the start of the field trial, with the inter-row distribution of compost (C) and pruning wood residues (PW), and the sowing of temporary cover crops (CC). White triangles indicate air sampling dates [**(A)** May 26, June 21, and July 26; **(B)** May 26, June 28, and July 24]. Black asterisks indicate the application of fungicide treatments followed disease-risk forecasts from vite.net^®^ (Horta s.r.l.; Rossi et al., 2012) for innovative inter-row treatments and white asterisks according to the fungicide schedule following the regional phytosanitary bulletins for the conventional inter-row treatment (PW). Black dots indicate tillage soil in PW and C inter-row treatments of tillage (T) soil regime. White dots indicate the CC mowing in both soil tillage regimes. Weather conditions: daily data of temperature (T; red line; in degrees Celsius), relative humidity (RH; green line; in percentage), rain (blue bars; in millimeters), and wetness duration (WD; light blue area; in hours).

Weather conditions varied markedly between the two sampling seasons,. In 2022, the average daily temperature was 22.7 °C (min of 8.3 °C, max of 31 °C), average relative humidity (RH) was 63%, and total rainfall during spore sampling periods (May-July) was 131.4 mm, distributed over 22 rainy days (**Figure 1A**). Leaf wetness totaled 16 h (**Figure 1A**), and winds mainly came from the southwest in May, from the east in June, and from the southeast in July, with mean wind speeds of 1.70, 5.45, and 2.27 m s^-^¹, respectively (**Supplementary Figure 1**). In 2023, the average temperature was slightly lower at 21.9 °C (min 7.6 °C, max 31.3 °C), RH increased to 67.2%, and total rainfall rose to 181.8 mm, distributed over 24 rainy days (**Figure 1B**). Leaf wetness was markedly higher, totaling 230 hours (**Figure 1B**), while the predominant wind directions shifted, with easterly winds dominating in May, westerly winds in June and southerly winds in July. Mean wind speeds for these months were 1.86, 2.25, and 2.05 m s^-^¹, respectively (**Supplementary Figure 1**). These inter-annual marked differences observed, likely influenced disease development, thus analyses of disease pressure and airborne microbial dynamics were conducted separately for each year.

### 3.2 Disease assessment

**Figure 2:**
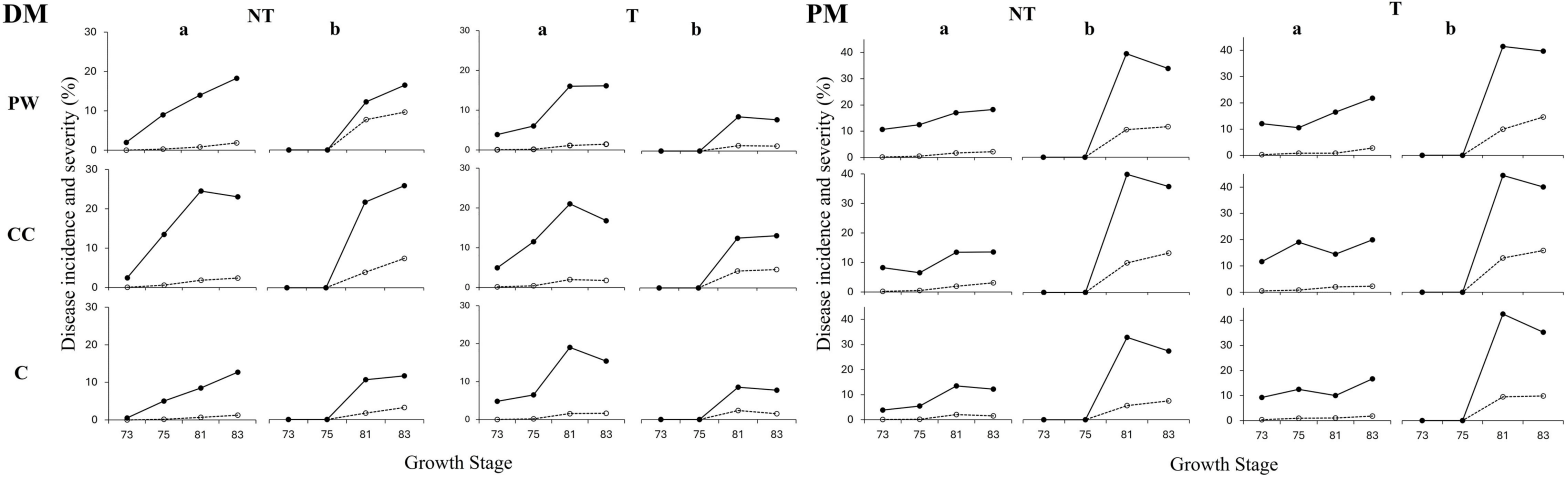
Downy (DM) and powdery (PM) mildew diseases incidence (% of affected leaves or bunch, black dots) and severity (% of affected leaf or bunch area, white dots) for leaf **(a)** and bunches **(b)** during the 2022 growing season in the tillage (T) and no-tillage (NT) regime and for each inter-row treatment (PW, Crumbling pruning wood; CC, Temporary Cover Crops; C, Compost). Data are averages of 3 replicates per each growth stage: Fruit Development (73: berries are groat-sized, clusters begin to hang and 75: pea-sized berries, with hanging clusters) and Berry Ripening (81: beginning of ripening, and 83: softening of berries).

The two seasons differed markedly in rainfall and temperature patterns, resulting in contrasting disease pressure and fungicide programs. A preliminary analysis revealed a significant plot × treatment × year interaction (data not shown); therefore, data were analyzed separately by year. The overall disease assessment for DM and PM showed that both were significantly higher in 2022 compared to 2023 (p < 0.001). During the first year (**Figure 2**), incidence and severity of downy mildew disease were generally mild (<3% and <0.15% respectively) at GS73, except for leaf incidence in the T regime (3.86–4.93%) where values were higher in the initial GS. During later GSs, leaf DM incidence and severity gradually increased in some treatments, such as PW and C in the NT regime; however, in other cases, peak values were observed at GS81 (e.g. CC treatment in both regimes). In contrast, bunches incidence and severity increased significantly at GS81, where disease levels remained relatively constant in the T regime but continued to increase in NT regime (**Figure 2**).

Regarding PM, during 2022 (**Figure 2**) the incidence and severity of the disease in leaves remained relatively constant values throughout the different GSs, while the incidence and severity in bunches fluctuated more markedly. The incidence of PM in bunches was 0% in GS73 and GS75, reaching a peak at GS81 (values between 32.5 and 44.5%). For PM bunch severity, the first GSs (73 and 75) showed no disease symptoms, and from GS81 onwards, severity values remained relatively stable (**Figure 2**).

DM during the second year showed consistently low incidence and severity (results reported in **Supplementary Figures 2A, B**). The same pattern was observed for BR disease in both years, with disease curves shown in **Supplementary Material** (**Supplementary Figures S2C, D)**.

**Figure 3:**
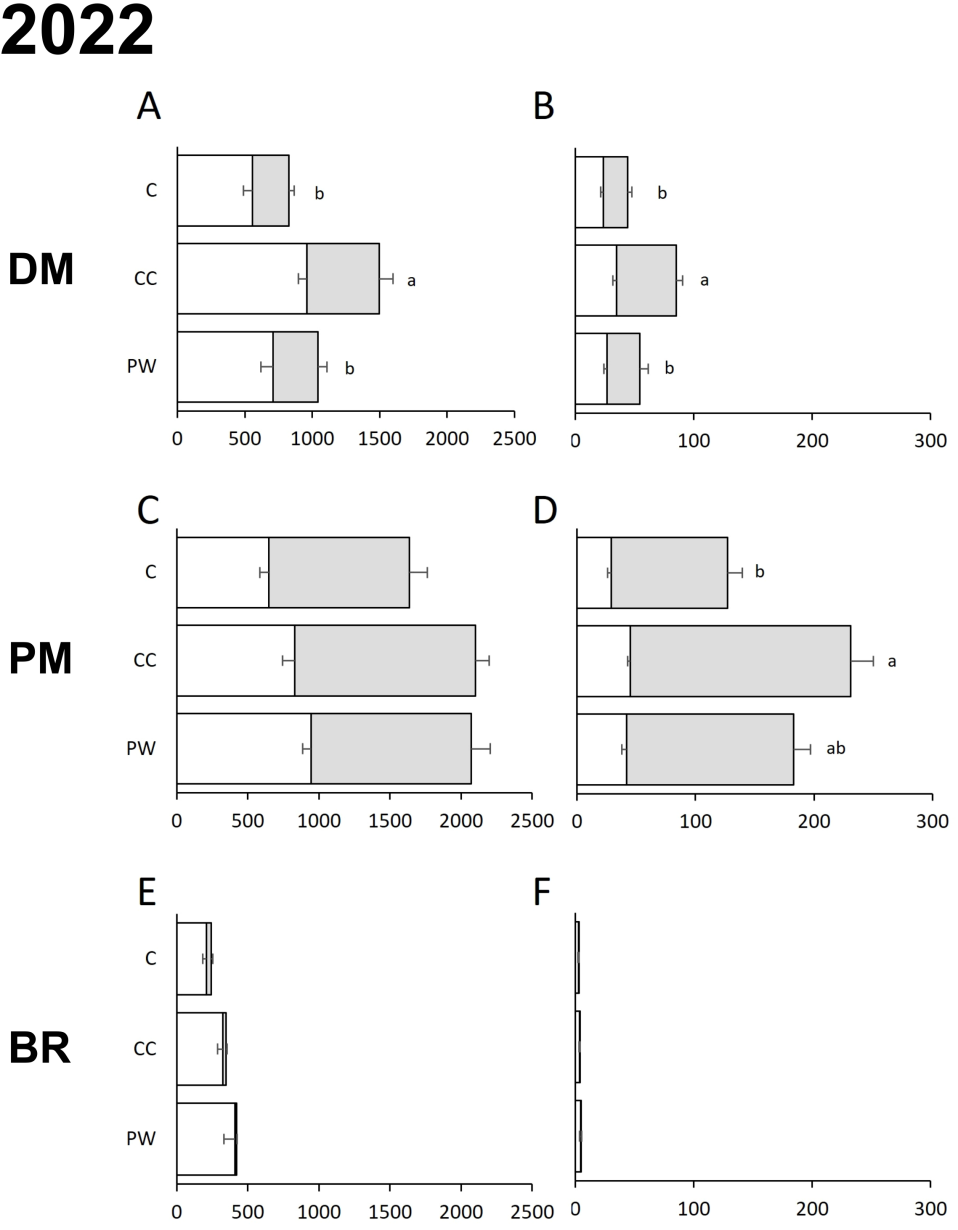
AUDPC calculated for incidence [**(A, C, E)** % of affected leaves, white bars, or bunches, grey bars] and severity [**(B, D, F)** % of affected leaves, white bars, or bunches, grey bars] for downy mildew [DM; **(A, B)**], powdery mildew [PM; **(C, D)**], and black rot [BR; **(E, F)**] during the 2022 growing season for each inter-row treatment (PW, Crumbling pruning wood; CC, Temporary Cover Crops; C, Compost). Data are averages of 3 replicates per year; whiskers are standard errors; AUDPC data were calculated by using the disease assessments at different plant growth stages: Inflorescence Emergence (55: Inflorescence swelling, flowers closely pressed together); Flowering (65: Full flowering, 50% of flowerhoods fallen); Fruit Development (73: berries are groat-sized, clusters begin to hang and 75: pea-sized berries, with hanging clusters); Berry Ripening (81: beginning of ripening, and 83: softening of berries). Letters show significant differences at the Tukey’s HSD test for overall data (AUDPC for leaves and bunches were summed up), with p=0.05.

**Figure 4:**
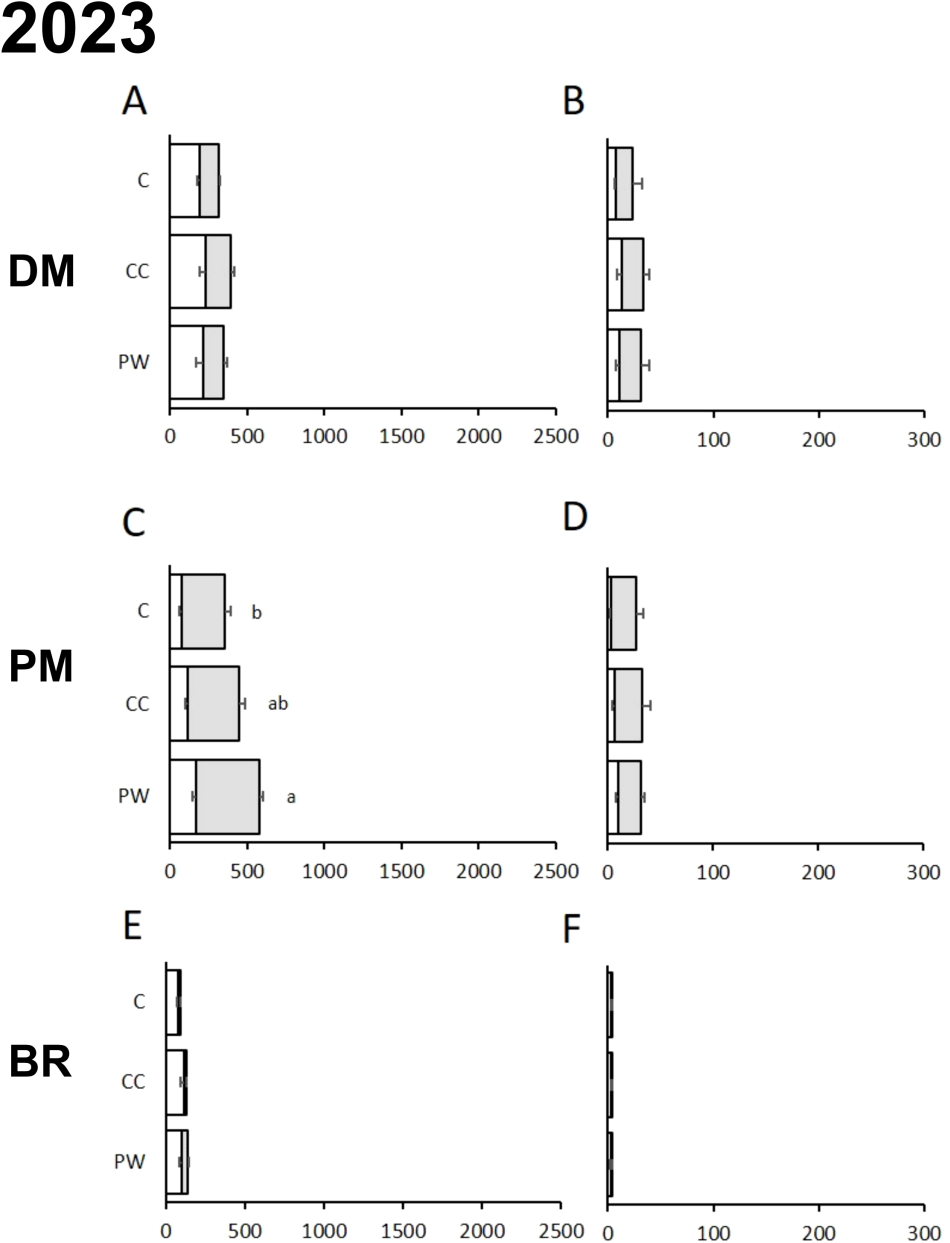
AUDPC calculated for incidence [**(A, C, E)** % of affected leaves, white bars, or bunches, grey bars] and severity [**(B, D, F)** % of affected leaves, white bars, or bunches, grey bars] for downy mildew [DM; **(A, B)**], powdery mildew [PM; **(C, D)**] and black rot [BR; **(E, F)**] during the 2023 growing season for each inter-row treatment (PW, Crumbling pruning wood; CC, Temporary Cover Crops; C, Compost). Data are averages of 3 replicates per year; whiskers are standard errors; AUDPC data were calculated by using the disease assessments at different plant growth stages: Inflorescence Emergence (55: Inflorescence swelling, flowers closely pressed together); Flowering (65: Full flowering, 50% of flowerhoods fallen); Fruit Development (73: berries are groat-sized, clusters begin to hang and 75: pea-sized berries, with hanging clusters); Berry Ripening (81: beginning of ripening, and 83: softening of berries). Letters show significant differences at the Tukey’s HSD test for overall data (AUDPC for leaves and bunches were summed up), with p=0.05.

In both years, the soil tillage regime significantly affected the AUDPC of PM calculated for the severity data on the leaves and bunches (**Table 2**). The effect of inter-row treatment was significant in 2022 for DM, affecting the AUDPC obtained from both incidence and severity data, and for PM, on severity only. Particularly, the use of compost (C) and crumbling pruning wood (PW) both reduced the AUDPC of DM calculated for both incidence and severity data compared to the use of temporary cover crop (CC) (**Figures 3A, B**); the inter-row treatment did not significantly affect the AUDPC for PM incidence (**Figure 3C**), while the AUDPC for PM severity was lower for C (**Figure 3D**). In 2023, the treatment affected only the AUDPC of PM for incidence data (**Table 2**; **Figure 4**), with a significant reduction for C. In both years, the interaction between soil tillage regime and inter-row treatment was not significant (**Table 2**). For BR, both factors did not affect the AUDPC for both disease and severity (**Table 2**).

**Table 2:** ANOVA results for the AUDPC calculated for incidence and severity data for downy and powdery mildews and black rot for soil tillage regime and inter-row treatment in 2022 and 2023.

Source of variation	df	Downy mildew				Powdery mildew				Black rot			
		Incidence		Severity		Incidence		Severity		Incidence		Severity	
		*P^1^*	%^2^	*p*	%	*p*	%	*p*	%	*p*	%	*p*	%
2022													
Soil tillage regime (1)	1	0.162	10.4	0.101	10.7	0.126	35.5	**0.033**	20.0	0.627	4.0	0.647	5.5
Inter-row treatment (2)	2	**0.006**	75.9	**0.001**	88.4	0.150	58.6	**0.002**	79.9	0.101	91.3	0.239	81.4
Interaction (1 x 2)	2	0.271	13.7	0.870	0.9	0.804	5.9	0.988	0.08	0.869	4.6	0.776	13.0
2023													
Soil tillage regime (1)	1	0.471	16.5	0.513	17.9	0.2857	7.8	**0.006**	66.8	0.765	1.4	0.895	0.9
Inter-row treatment (2)	2	0.419	55.7	0.679	31.4	**0.0105**	85.2	0.648	5.4	0.244	49.4	1.000	0.0
Interaction (1 x 2)	2	0.638	27.8	0.542	50.7	0.5878	6.9	0.140	27.8	0.246	49.1	0.418	99.0
^1^ p-value; ^2%^ of variance													

Incidence was considered the sum of the AUDPC of affected leaves and bunches, and severity was considered the sum of the AUDPC of affected area of leaves and bunches.

Bolded values indicate significance at p < 0.05.

### 3.3 Harvest

Bunch number per vine did not differ among treatments in either season. In 2022, bunch weight was significantly higher under no-till (NT) than tillage (T) regimes (3.84 kg vs. 2.31 kg; **Table 3**, p < 0.001). The inter-row treatment also had a significant impact, with higher bunch weight observed in vines managed with C and PW (p < 0.05); however, the interaction between tillage and inter-row treatment was not significant. In 2023, both the soil tillage regime (*p* < 0.01) and the interaction between regime and inter-row treatment (p < 0.05) significantly influenced bunch weight, with the highest bunch weight (7.12 kg) recorded under the full conventional management, represented by the T regime with PW inter-rows treatments (**Table 3**). The °Brix values were significantly higher in the T and CC combination (23.96; p < 0.05; **Table 3**).

**Table 3:** Average number, weight, and °Brix of bunches at harvest for each soil tillage regime (T, tillage and NT, not tillage) and inter-row treatment (CC, Temporary Cover Crops; C, Compost; PW, Crumbling pruning wood) in 2022 and 2023.

Yield and quality parameters	Soil tillage regime	2022				2023			
		Inter-row treatment				Inter-row treatment			
		CC	C	PW	Average	CC	C	PW	Average
Number	T	40.00	36.80	40.30	39.03	22.50	21.80	24.40	22.32
	NT	43.90	46.80	44.40	45.03	20.00	22.10	23.10	21.73
	Average	41.95	41.80	42.35	42.03	21.25	21.95	23.75	22.32
Weight (Kg)	T	1.83	2.63	2.47	2.31^B^	4.47^b^	3.68^b^	7.12^a^	5.09
	NT	2.69	4.57	4.25	3.84^A^	5.25^ab^	4.04^b^	4.88^ab^	4.72
	Average	2.26^b^	3.60^a^	3.36^ab^	3.07	4.86^ab^	3.86^b^	6.00^a^	4.91
°Brix	T	–	–	–	–	23.96^a^	23.70^ab^	22.86^c^	23.51
	NT	–	–	–	–	23.13^bc^	23.23^abc^	23.86^ab^	23.41
	Average	–	–	–	–	23.55	23.46	23.36	23.46

Letters show significant differences at the HSD test (*p* < 0.05). Differences between regimes are indicated with capital letters (A, B); differences between treatments are indicated with small bold letters (a, b, c), differences in the interactions are indicated with small letters (a, b, c).

### 3.4 Airborne microbiome biodiversity

**Figure 5:**
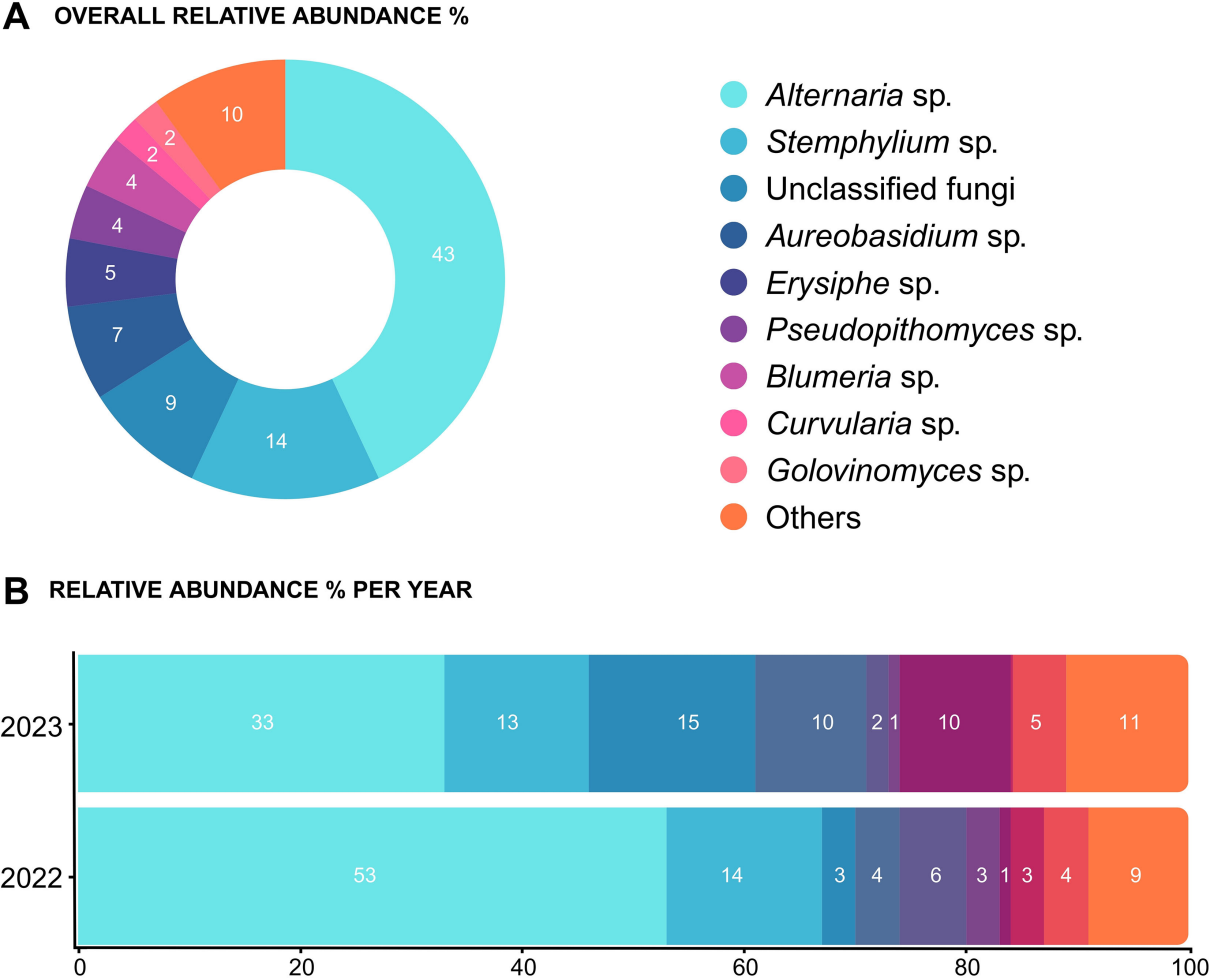
Overview of the main genera relative abundance (%) detected by the airborne microbiome sampling conducted with the spore sampler **(A)**. Differences among the two sampling years regarding the main taxa relative abundances are represented by hierarchical clustering **(B)**. Only the most abundant taxa are displayed, while the remaining less abundant taxa are grouped into the category “Others”.

**Figure 6:**
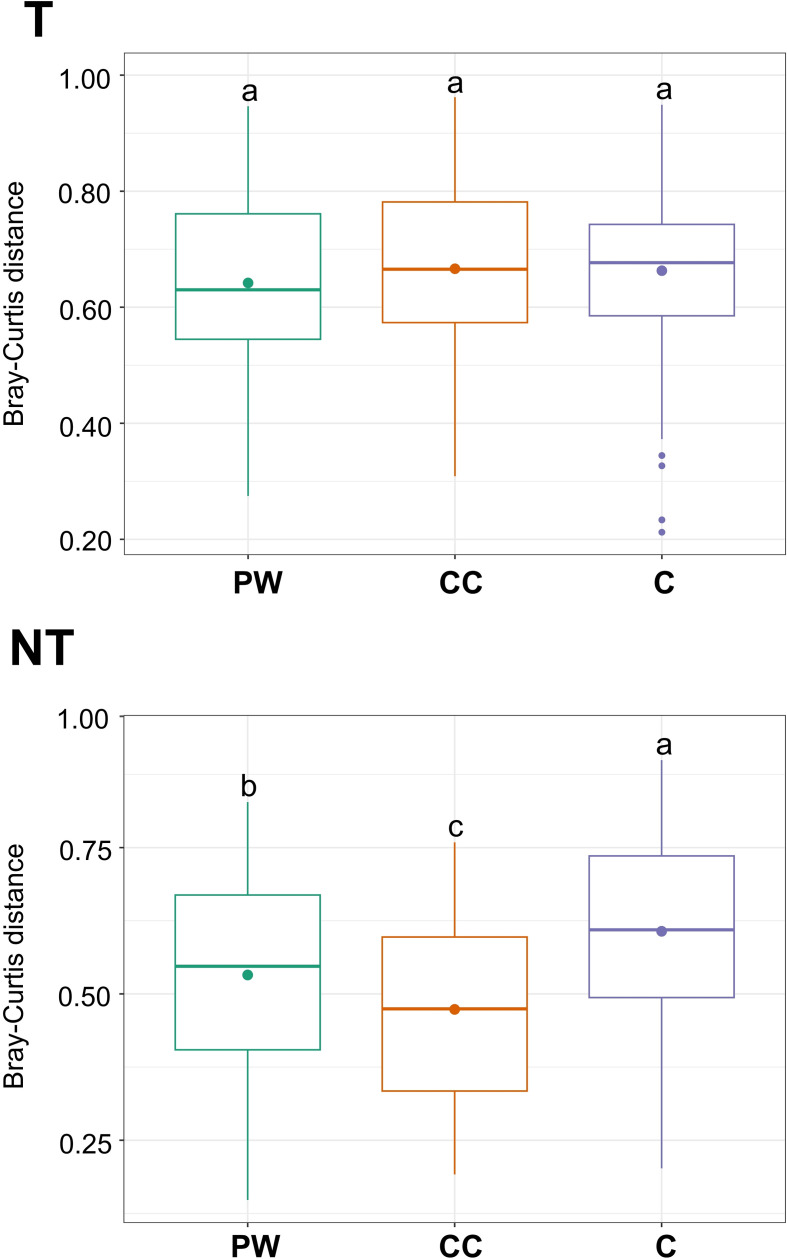
Fungal β-diversity across cover crop treatments under Tillage (T) and No-Tillage (NT) soil regimes. β-diversity was evaluated using Bray–Curtis dissimilarity, calculated by merging data from multiple sampling months and years to capture overall temporal trends across the two sampling seasons. Bars represent mean β-diversity within each treatment, and different letters indicate statistically significant differences between treatments (p < 0.05).

After quality control and data pre-processing, a total of 982 ASVs were retained from the original set of 1,529 ASVs. The total number of reads after processing was 3,767,762 with an average of 34,886 reads per sample. The fungal genera with the highest relative abundances detected in both years are shown in **Figure 5**. Across seasons, α-diversity differed by year (Chao1 *p* < 0.05; Shannon *p* < 0.05; Observed *p* < 0.01; **Supplementary Figure 3** upper panel) and β-diversity also varied (PERMANOVA R² = 0.119, *p* < 0.001; **Supplementary Figure 3** lower panel). Overall β-diversity patterns across the experiment were assessed using Bray-Curtis dissimilarity to compare fungal community composition among the two tillage regimes (T and NT) and the three inter-row treatments (C, CC, and PW), excluding the sampling month factor. Results obtained in each sampling month are reported in **Figure 6**. Under tillage (T) regime, β-diversity did not differ significantly among inter-row treatments (**Figure 6**). Conversely, under the no-tillage (NT) system, cover crop management had a more pronounced effect on the aerial microbiota. Specifically, compost (C) plots showed the greatest Bray-Curtis dissimilarity relative to other treatments (**Figure 6**, *p* < 0.05).

**Figure 7:**
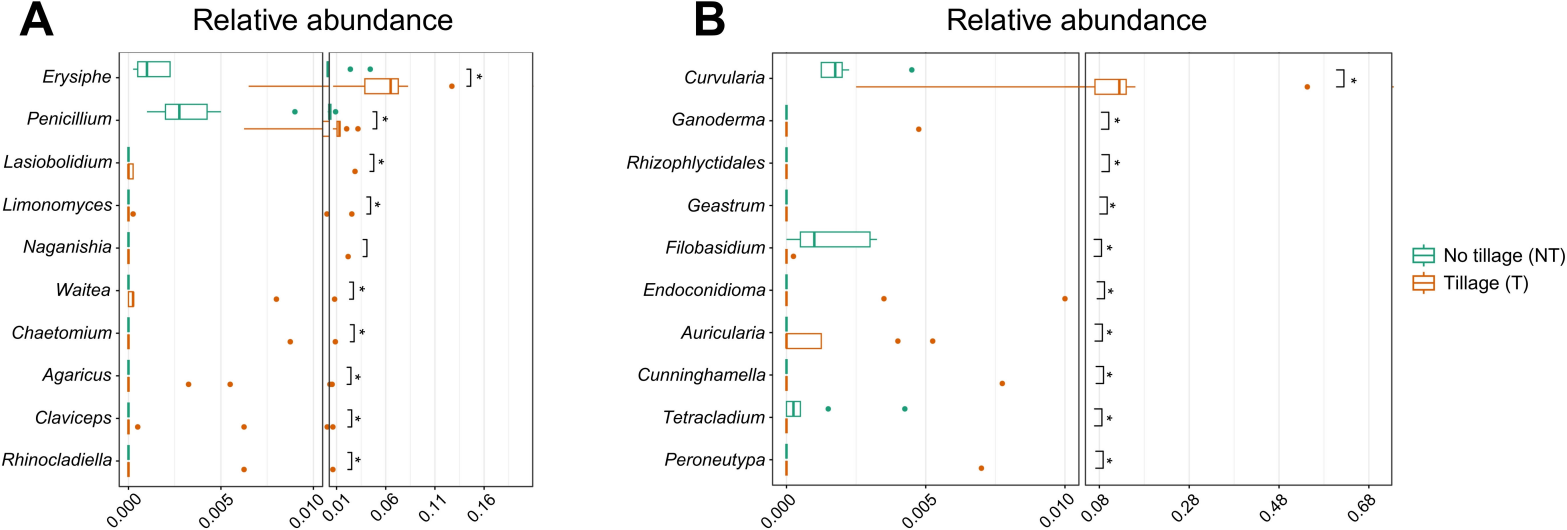
Graphical summary of Metastats analysis showing the relative abundance of the top 10 fungal genera in the aerial microbiota collected under tillage (T, orange bars) and no tillage (NT, green bars) soil regimes in June **(A)** and July **(B)**, 2022. Boxplots display the distribution of relative abundance for each genus, with individual points representing specific sample values. Single asterisk (*) Indicates statistical significance at p<0.05.

In 2022, α-diversity varied significantly between sampling months only for the Chao1 estimator (*p* > 0.05; data not shown), while sampling month was also a significant source of variation in β-diversity (R^2^ = 0.201, *p* < 0.001; data not shown). The dominant airborne fungal genera in May were *Alternaria* (41.9%), *Blumeria* (15.9%), *Stemphylium* (14.5%), and *Golovinomyces* (8.9%). In June, *Alternaria* increased in relative abundance to 59.6%, while *Stemphylium* decreased slightly to 11.2%. *Aureobasidium* and *Pseudopithomyces* were also detected at 4.0% and 4.3%, respectively. In July, *Alternaria* (53.1%) and *Stemphylium* (15.7%) remained dominant, followed by *Curvularia* (7.6%), *Aureobasidium* (6.6%), *Pseudopithomyces* (5.5%), and *Rhizopus* (5.3%) (**Supplementary Figure 4**). In May 2022, α-diversity did not significantly differ between soil tillage regime (T vs. NT) or among inter-row treatments (PW vs. CC vs. C) for any estimator (Chao1, Shannon, Observed). However, β-diversity analysis indicated a significant difference in community composition between soil tillage regime (R² = 0.039, p = 0.031), although no differences were detected among inter-row treatments. LEfSe analysis identified eight genera as significantly different between soil tillage regime (**Supplementary Figure 6**). In June 2022, α-diversity was significantly different only for the Shannon index, with higher diversity observed in the tilled regime (p < 0.01; data not shown). No significant α-diversity differences were found among inter-row treatments. β-diversity analysis confirmed that fungal community composition differed significantly between T and NT (R² = 0.112, p = 0.040). LEfSe identified 11 differentially abundant genera, including *Erysiphe*, which appeared to be enriched in T regime (**Supplementary Figure 6**). A Metastats model was applied to confirm and quantify this difference. Metastats results confirmed *Penicillium* (p < 0.001) and *Erysiphe* (p = 0.001) as the most significant genera distinguishing T from NT regime, with LDA scores of -3.74 and -4.43, respectively (**Figure 7A**). In July 2022, α-diversity indices revealed a significant difference between soil tillage regime only for the Observed species index (p = 0.008; data not shown), with higher species richness observed in the NT soil regime. No significant differences in α-diversity were found between inter-row treatments. β-diversity analysis again showed a significant difference between soil tillage regime (R² = 0.140, p = 0.025), but not among inter-row treatments. LEfSe identified 14 differentially abundant genera between T and NT (**Supplementary Figure 6**). Metastats analysis detected four genera as primary contributors to differences between soil tillage regime with *Curvularia* (p = 0.002) enriched in T and *Filobasidium* (p < 0.001) enriched in NT (**Figure 7B**).

In 2023, the α-diversity of fungal communities was significantly different between sampling months only for the Shannon index (p =0.001), indicating differences in community evenness across the growing season. Sampling month was also a significant source of variation in β-diversity (R² = 0.297, p = 0.001; data not shown), suggesting that the overall composition of the airborne fungal community changed over time. The most abundant airborne fungal genera in 2023 also varied across sampling months (**Supplementary Figure 5**). In May, the dominant genus was *Alternaria* (27.3%), followed by *Aureobasidium* (3.5%), *Blumeria* (3.5%), and *Erysiphe* (0.5%). In May, 34.8% of reads were not taxonomically categorized. In June, *Alternaria* increased to 38.8%, while *Aureobasidium* and *Erysiphe* abundance increased sharply to 17.6% and 10.2%, respectively, indicating a substantial shift in the fungal community structure. In July, *Alternaria* slightly decreased to 31.9%, while *Erysiphe* increased further to 18.9%, becoming the second most abundant genus. Meanwhile, *Aureobasidium* declined to 7.4%, and other genera such as *Curvularia* and *Blumeria* remained consistently present at lower relative abundances throughout the season. In May 2023, α-diversity did not differ significantly between soil tillage regime (T vs. NT) or among inter-row treatments (PW, CC, C). Similarly, β-diversity analysis revealed no significant differences in community composition between either factor. In June 2023, as in May, α-diversity showed no significant differences between soil tillage regimes or inter-row treatments. However, β-diversity indicated a significant difference between T and NT regimes (R² = 0.111, p = 0.028), but not among inter-row treatments. Metastats analysis identified five genera as significantly contributing to the dissimilarities between soil tillage regimes (**Supplementary Figure 7**). Finally, in July 2023, α-diversity indices showed significant differences between soil tillage regimes for both the Shannon and Observed indices (p = 0.0096 and p = 0.0356, respectively), with higher diversity observed in the tilled regime. No significant α-diversity differences were found in inter-row treatments. For β-diversity, no significant differences were observed between tillage regimes, but a significant difference was detected among inter-row treatments (R² = 0.263, p = 0.002). LEfSe analysis identified 11 genera as key contributors to the dissimilarities between inter-row treatments (**Supplementary Figure 7**).

## 4 Discussion

This study evaluated how different inter-row treatments, combined with contrasting soil tillage regimes, shaped the vineyard air-borne fungal community across two growing seasons (2022 and 2023) and how these dynamics related to disease incidence and severity. Two main patterns emerged. Taken together, these results suggest that soil tillage acts as a primary ecological filter for the airborne fungal community, influencing it through a combination of physical disturbance, which promotes spore release and resuspension near the soil surface, and ecological filtering, by altering habitat and microenvironmental conditions. This process promotes the release and redistribution of soil- and residue-associated taxa, including key grapevine pathogens. In contrast, inter-row management practices appear to modulate this airborne assemblage only under specific environmental conditions, with their effects being readily overridden under high disease pressure and prolonged wetness. First, compost (C) and pruning-wood (PW) mulches reduced downy (DM) and powdery (PM) mildews in 2022 but their effect was not detectable in the wetter 2023 season. Second, soil tillage regime (tillage, T, vs. no-tillage, NT) showed a relatively stronger influence on airborne fungal community than inter-row treatments, particularly during mid-season peaks, although the overall proportion of explained variance remained limited. In contrast, low emergence of selected cover crops (CC) and prevalence of spontaneous flora likely reduced the potential barrier effect of CC in 2022. It should be noted that sampling dates were selected to coincide with key grapevine phenological stages and periods of higher disease pressure. While this approach allowed us to focus on biologically relevant phases of vineyard disease dynamics, it may limit the interpretation of airborne microbiome shifts across the entire growing season.

The composition of air-borne fungal population surrounding vineyards has previously been studied with culturing approaches, showing that air microbial communities are strongly influenced by environmental variables, such as rainfall, sunlight hours, and the number of dry days (Magyar et al., 2009). Other studies have highlighted links between airborne and vine-associated microbiome (Pérez-Martín et al., 2014). For example, Abdelfattah et al. (2019) demonstrated that a portion of the airborne microbiota contributes to shaping the vine-associated microbial community. Unlike earlier methods relying on Vaseline-coated spore traps left in the field, which may introduced bias toward certain taxa, our real-time vacuum sampling minimized potential selection during sampling, capturing the airborne community with greater precision. The present study focused on fungal populations only because preliminary trials confirmed that bacterial populations were not detectable using 16S V3-V4 primers with the captaspore sampling, while fungal populations were consistently detectable using ITS1–2 primers. Oomycetes population could not be assessed with this approach as their characterization requires a different set of primers (Riit et al., 2016). For this reason, we focused our study on fungal populations only.

In 2022, C and PW treatments reduced DM incidence and severity and PM severity. The hypothesis is that C acted as a physical barrier, blocking spores’ dispersal from soil to canopy. This mechanism is consistent with prior studies showing that residues on soil surface (e.g., cut cover crops left in the inter-row) reduce pathogen dispersal (Fitt et al., 1988; Ristaino et al., 1997; X. Yang et al., 1990). No effect was observed in CC treatment probably for the low level of emergence of selected CC, caused by drought in the experimental season. Black rot (BR) was not significantly influenced by any inter-row treatment, probably due to low disease pressure.

In 2023, C reduced PM incidence, supporting its function as a physical barrier in the dispersion of spores. Favorable weather conditions and consequent higher disease pressure required more fungicide applications, which likely masked treatment effects on disease epidemics. This context prevents us from drawing conclusions about the effect of soil management practices on disease management. Although different fungicide application schedules were followed between conventional and innovative treatments, the high pressure of the disease in the second year meant that both systems were similar in terms of number of applications during the cycle. These outcomes align with other studies reporting reduced disease severity in cover-cropped plots, but less so when fungicides were also applied, probably due to the low incidence of diseases (Hasanaliyeva et al., 2024).

In both years, CC reduced bunch weight but increased must °Brix, likely due to competition for water and nutrients between the cover crop and the main crop (Celette and Gary, 2013; Garcia et al., 2018). Unlike typical management practices where CC is mowed at flowering, the inter-row vegetation was cut later, around grape ripening, to allow sufficient cover establishment. This may have impaired competition but simultaneously promoted sugar accumulation, a potential advantage in terms of wine production, depending on the oenological objectives, where higher sugar values improve grape composition (Muscas et al., 2017; Wang et al., 2022).

Microbial diversity analyses further highlighted management effects. In different sampling months over the two years, T increased α-diversity (Shannon’s index), indicating greater genera and community diversity in T compared to NT soil regime, contrary to expectations that NT would harbor greater fungal diversity. It is generally agreed that NT increases soil bacterial diversity, but fungal responses are more variable (Dong et al., 2017). β-diversity was different among soil tillage regimes and inter-row treatments, with lack of consistency. This trend was also observed in the study of Bansal et al. (2024) who tested different combinations of soil tillage and grazing with sheep in the inter-row vineyard, detecting precise results for the bacterial community but they did not see clear effects on fungal microbiome. Homogeneity of dispersion tests indicated that for cover crops (CC), NT and T groups differed significantly in within-group variability (p < 0.001), whereas pruning-wood (PW) and compost (C) treatments did not. This suggests that some of the β-diversity differences observed for CC may reflect variation in dispersion rather than shifts in community centroids alone. Therefore, while CC influenced the airborne fungal community, these results should be interpreted cautiously, acknowledging that within-group heterogeneity may partly drive the observed PERMANOVA differences. Overall, T was associated with greater community differentiation than inter-row treatment, and under NT, compost increased β-diversity, while CC reduced it compared to PW, although the magnitude of this effect was modest. These results suggest that organic amendments such as compost may play a stronger role in shaping the aerial fungal communities, while in tilled systems, the microbial component of the so-called *terroir*, specifically its aerial fraction (Magyar et al., 2009), may be more resilient to mulch-induced changes.

Indicator taxa reflected both management and disease relevance. *Penicillium*, *Erysiphe*, *Curvularia*, and *Malassezia* were more frequently associated with T regimes, a pattern that can be explained by the disturbance-driven release of organic matter and litter residues, which favors the proliferation, release, and dispersal of the obligate biotroph *Erysiphe* and fast-growing fungi like *Penicillium* (Korniłłowicz-Kowalska et al., 2022). It should be noted that these associations are suggestive and do not establish direct causal links between soil management practices and disease development, as airborne abundance alone does not necessarily translate to infection in grapevines. Generally, *Penicillium* is a large ubiquitous genus, and represents the most abundant taxa of fungi in air and soils (Yadav et al., 2018). Among the diverse ecological functions of *Penicillium*, its role in decomposition is noteworthy (Rodriguez Assaf et al., 2020). *Erysiphe* includes numerous plant pathogen species that form white, powdery dust on the vegetative and reproductive parts of flowering plants (Taylor et al., 2015). Within this genus is the species *E. necator*, important in grapevines, as it is the causal agent of powdery mildew (Gadoury et al., 2012). The Metastats also detected *Curvularia* and *Malassezia*, both stress-adapted fungi, as the most important genera that distinguished T soil regime but their pathogenic relevance in grapevine remains limited; they may also benefit from the altered soil-plant-air interface of tilled regimes (Sharma-Poudyal et al., 2017). Conversely, *Diplodia* and *Filobasidium* were the most important genera associated to the NT regime, in July 2022. NT practices are known to preserve surface residues and maintain higher moisture, conditions that support wood decomposers such as *Diplodia*. However, the genera comprise pathogenic species for grapevine like *D. corticola* causing cankers in the vascular tissue (Úrbez-Torres et al., 2010) or *D. seriata* responsible of dieback disease (Larach et al., 2024). Similarly, yeasts like *Filobasidium* were also detected in high abundance in NT and their presence can be linked to the more humid microhabitats created by the reduced soil disturbance (Frøslev et al., 2022).

Finally, among the genera that characterized 2022 and 2023 sampling years, we observed an abundant presence of *Aureobasidium*, a genus traditionally known for its biocontrol activity against *Botrytis cinerea*, the causa agent of bunch rot in grapevines (Parafati et al., 2015). The progressive increase in *Aureobasidium* abundance across sampling months and years may suggest a seasonal reinforcement of natural biocontrol potential. In 2022, *Aureobasidium* was not detected in May, and appeared during June and July at relative abundances of 4 and 6%, respectively. In contrast, during the 2023 season, the genus was already present in May (3%), increases to 7% in June, and reached a no**Table 2**1% in July. This seasonal increase in *Aureobasidium* abundance is likely linked to the increment of vegetal material across the two seasons and to the accumulation of senescing tissues and exudates during berry maturation in the same season (Costantini et al., 2022). Its ecological versatility and strong adaptation to the vineyard phyllosphere enabled it to persist and proliferate throughout the seasonal shifts in temperature and rainfalls to which other taxa can be more subjected (Parafati et al., 2015).

## 5 Conclusion

This study was designed as a mechanistic case study rather than a large-scale validation trial and demonstrates that inter-row soil management and tillage can influence both aerial fungal biodiversity and grapevine disease development. While we observed associations between soil management practices and potentially pathogenic taxa such as Erysiphe and Diplodia, these trends are suggestive and do not establish causal links to disease development. Our findings indicate that compost mulching significantly reduced DM and PM under moderate disease pressure but was less effective under wetter conditions, highlighting the dominant influence of seasonal weather. Tillage affected fungal community composition more strongly than inter-row cover treatments, with NT regimes showing greater sensitivity to organic amendments. While cover crops reduced yield, they enhanced sugar concentration, providing a potential tool for modulating grape composition. The high-efficiency spore vacuum sampler also proved valuable for accurately characterizing airborne fungal communities and sensitively detecting pathogens. Collectively, these findings support the use of compost mulches to lower disease pressure in drier seasons, their integration with fungicides under high-risk conditions, and minimal tillage to stabilize airborne fungal communities while limiting pathogen inoculum. Longer-term and larger-scale studies linking airborne diversity to vine-associated microbiomes will be essential for developing robust, ecosystem-specific IPM strategies in vineyards.

## Data Availability

The datasets presented in this study can be found in online repositories. The names of the repository/repositories and accession number(s) can be found in the article/**Supplementary Material**.
