# Supplementary material for: Soil management modulates vineyard airborne fungal communities and impacts fungal disease pressure
Source: Front Plant Sci. 2026 Apr 10;17:1770877. doi: 10.3389/fpls.2026.1770877 (PMC13106304; doi:10.3389/fpls.2026.1770877)
Supplement: Supplementary file 1 [file DataSheet1.pdf]

# Supplementary material

# Soil management modulates vineyard airborne fungal communities and impacts fungal disease pressure

**Florencia Asinari[1,3]†, Gabriele Bellotti[2]†, Giorgia Fedele[1,4], Edoardo Puglisi[2*], and Tito Caffi[1,4]**

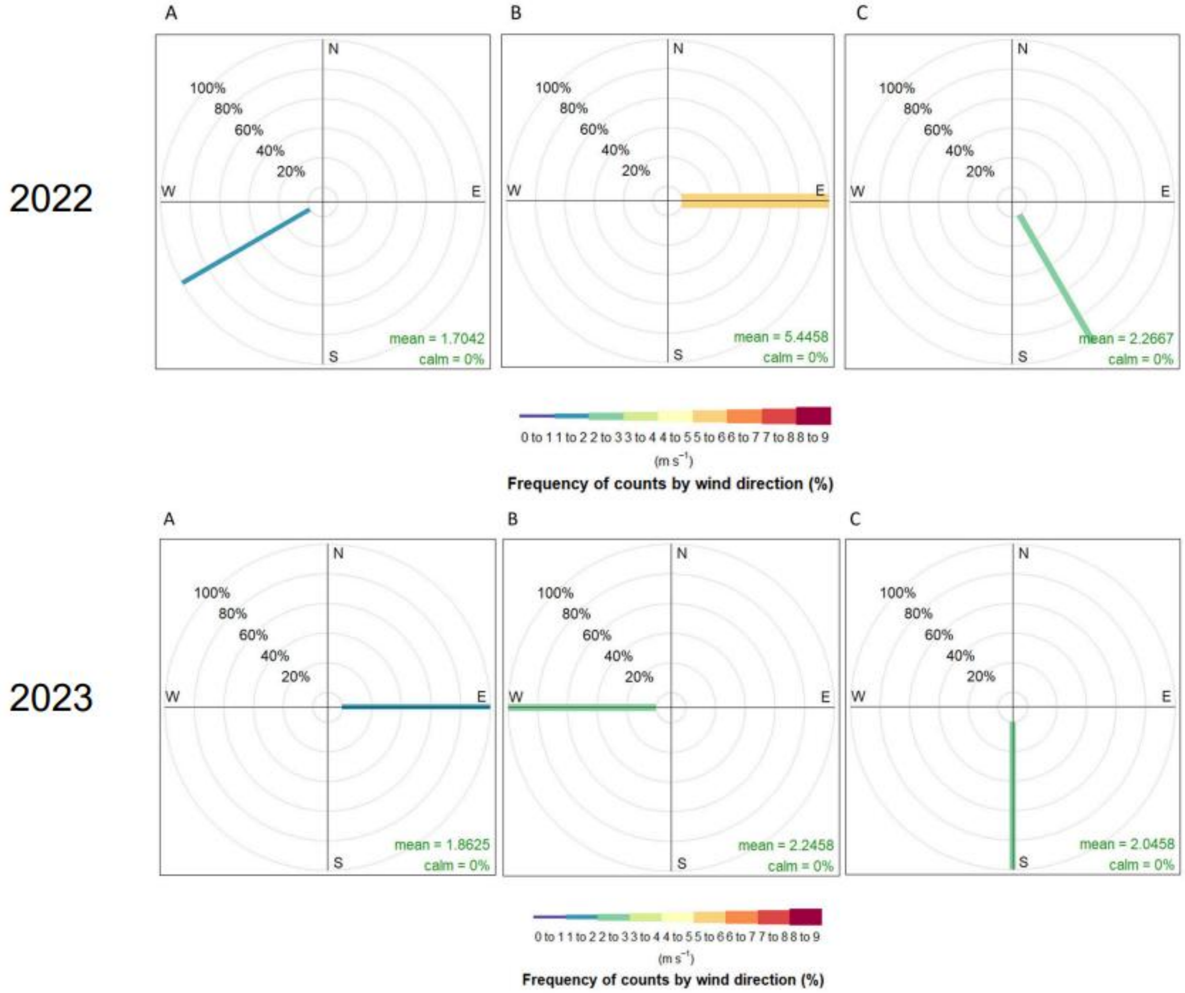


**Supplementary Figure 1.** Wind rose diagrams showing the frequency of wind direction and associated wind speed classes (m $s^{-1}$) recorded during the 2022 and 2023 growing seasons at Gasparini experimental vineyard of the Università Cattolica del Sacro Cuore, Italy. Sampling date in 2022 (**A**. May 26; **B**. June 21 and **C**. July 26) and 2023 (**A**. May 26; **B**. June 28 and **C**. July 24).

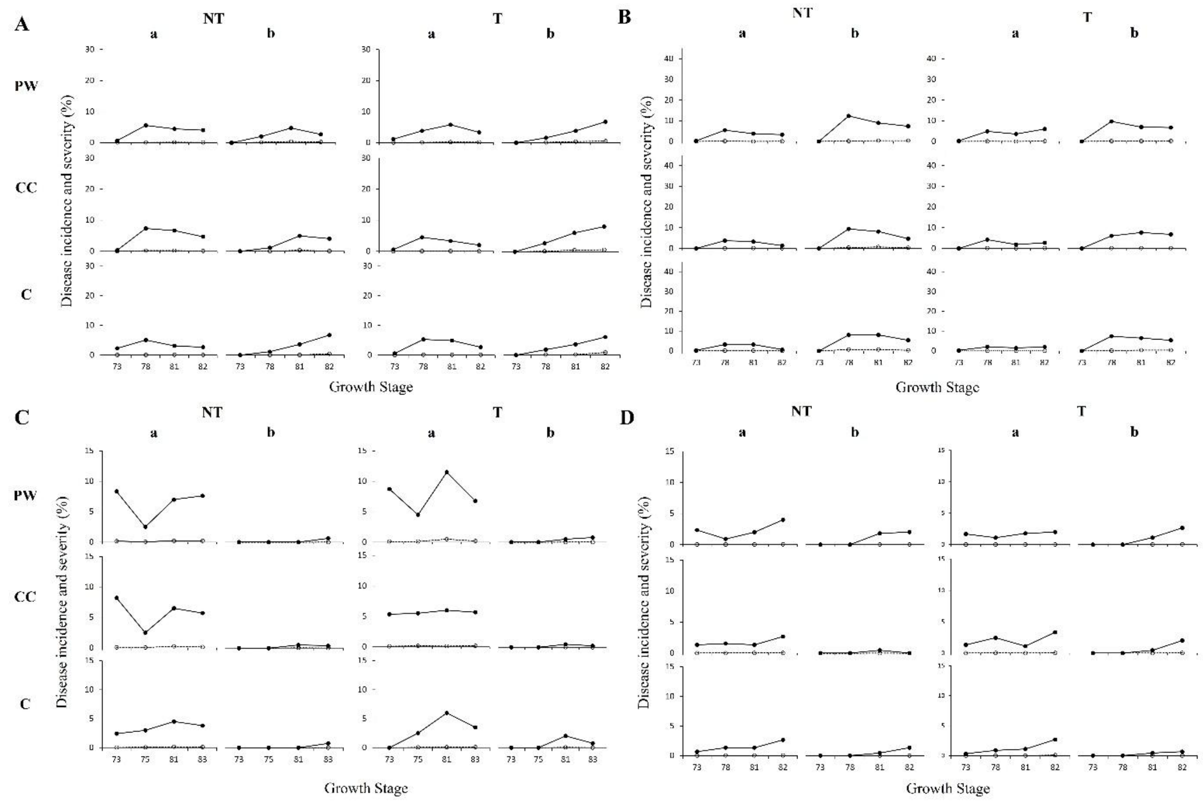


**Supplementary Figure 2.** Diseases incidence (% of affected leaves or bunch, black dots) and severity (% of affected leaf or bunch area, white dots) for leaf (a) and bunches (b) in the tillage (T) and not tillage (NT) systems and for each strategy (PW: Crumbling pruning wood, CC: Temporary Cover Crops, C: Compost). **A.** Downy mildew disease during 2023 growing season. **B.** Powdery mildew disease during 2023 growing season. **C.** Black rot disease during 2022 growing season. **D**. Black rot disease during 2023 growing season.

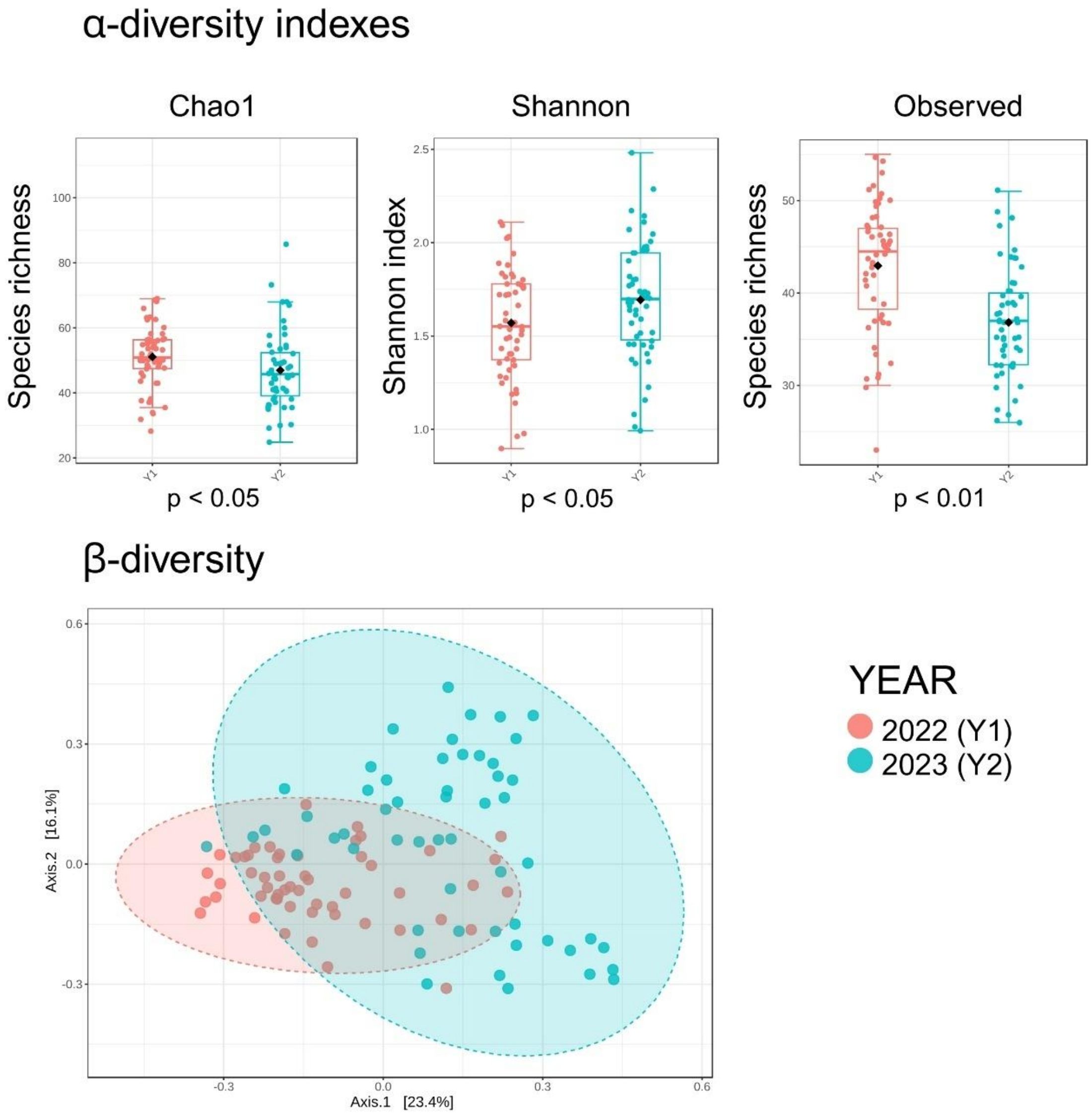


**Supplementary Figure 3.** α-diversity and β- diversity indexes illustrating differences in the fungal communities in air spore samples collected in 2022 (red, Y1) and 2023 (light blue, Y2). **Upper**, the α-diversity based on Chao1, Shannon, and Observed diversity indicators represented by boxplot. The box extends from the 25th to the 75th quartile of the data distribution, the line crossing the box represents the median, and the black diamond indicates the average; whiskers extend to the maximum and minimum. **Lower**, Principal Coordinate Analysis (PCoA) based on Bray–Curtis dissimilarity metrics, showing the distance in the β-diversity of fungal communities present in air spore samples collected in 2022 and 2023. Areas show distinct clustering of 2022 (red) and 2023 (lightblue).

**Supplementary Table 1**. Pairwise PERMANOVA analysis of β-diversity between 2022 (Y1) and 2023 (Y2) samples. The table reports the F statistic, $R^2$ (proportion of variance

explained), p-values, and Benjamini–Hochberg adjusted p-values (p.adj). Significant results are indicated by an asterisk (*).

| Pairwise permanova | F.value | R.squared | p.value | p.adj |
|---|---|---|---|---|
| Y1 .vs. Y2 | 14.29 | 0.1188 | 0.001* | 0.001* |

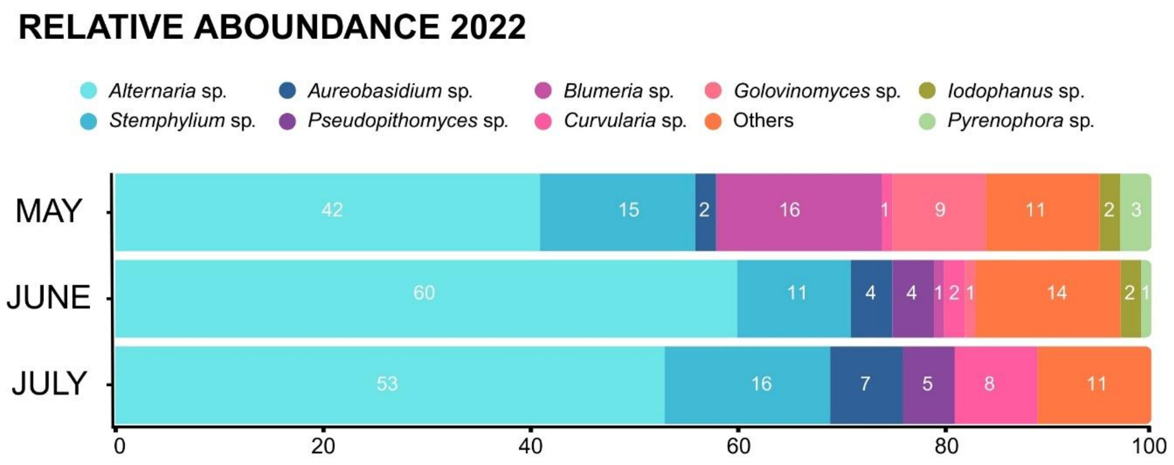


**Supplementary Figure 4.** Differences among sampling months detected during 2022 regarding the main taxa relative abundances are represented by the hierarchical clustering. Only the most abundant taxa are displayed, while the remaining less abundant taxa are grouped into the category "Others."

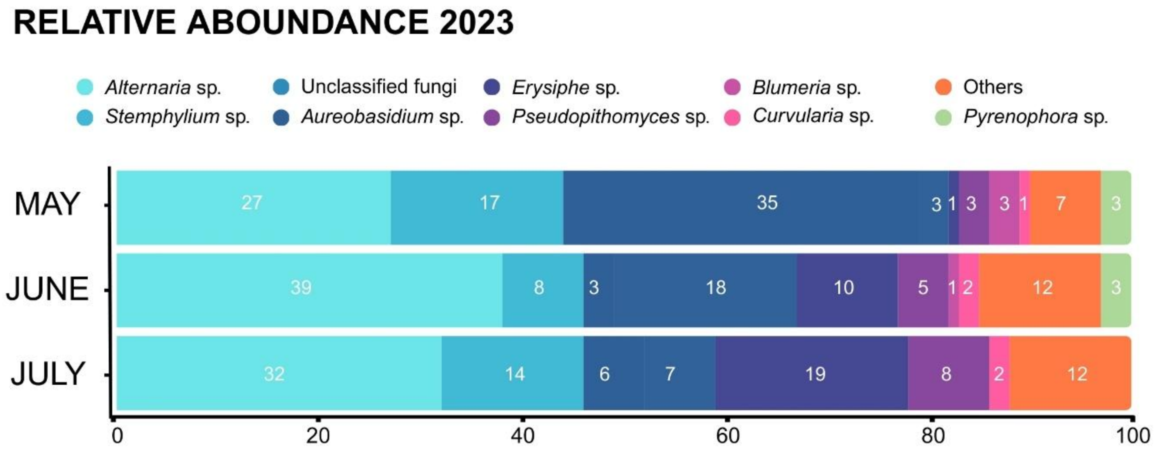


**Supplementary Figure 5.** Differences among sampling months detected during 2023 regarding the main taxa relative abundances are represented by the hierarchical clustering. Only the most abundant taxa are displayed, while the remaining less abundant taxa are grouped into the category "Others."

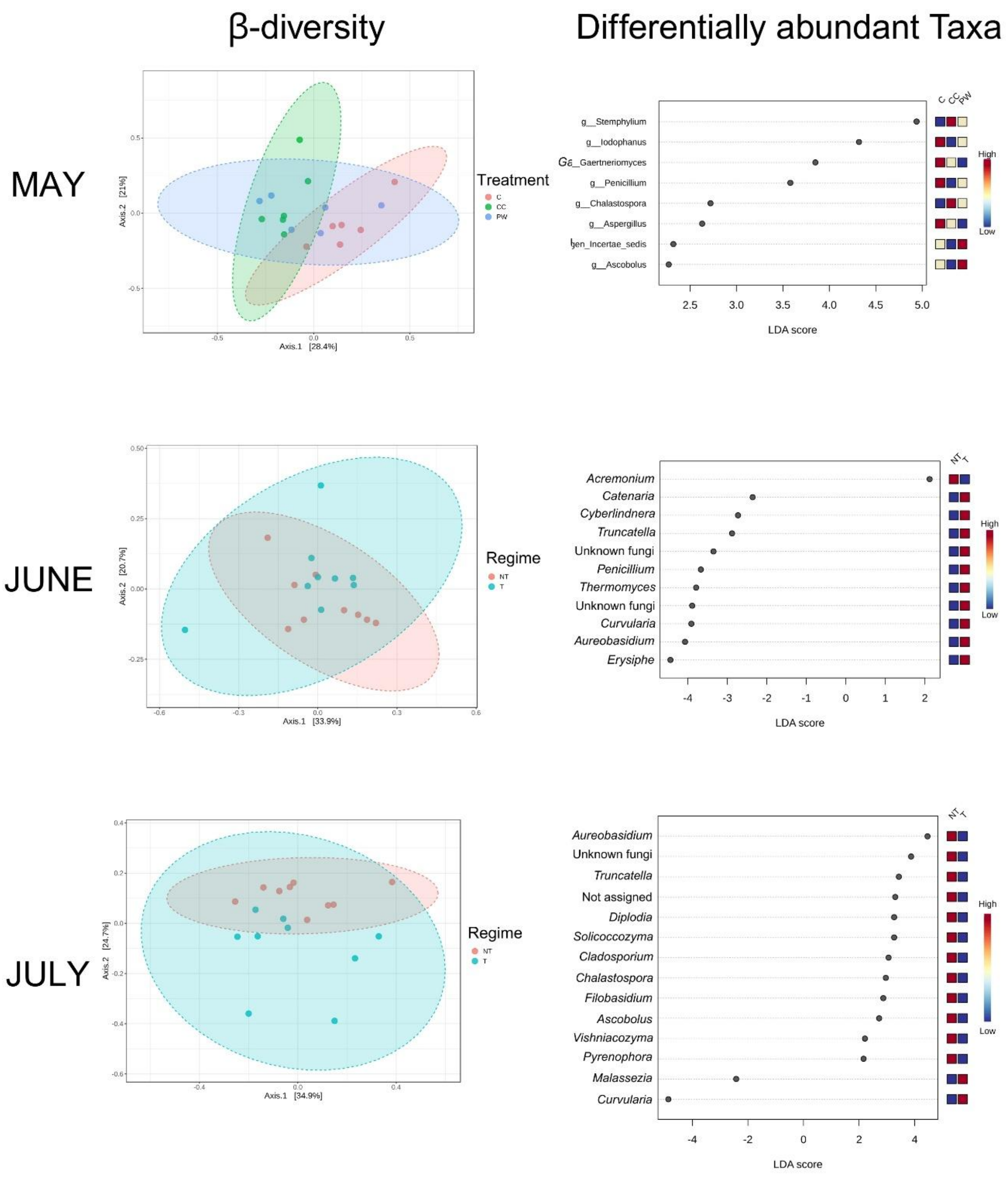


**Supplementary Figure 6.** Fungal β-diversity and differentially abundant taxa across sampling months in 2022. Principal Coordinate Analysis (PCoA) based on Bray-Curtis dissimilarity illustrates the fungal community β-diversity that was significantly different for June and July. LEfSe analysis highlights the fungal taxa that underwent significant shifts each month in relation to tillage in June and the soil management practice applied: cover cropping in July.

**Supplementary Table 2.** Pairwise PERMANOVA analysis of β-diversity among experimental groups (C, CC, PW) for samples collected in May 2022. Analyses were performed using 999 permutations. The table reports the F statistic, $R^2$ (proportion of variance explained), p-values, and Benjamini–Hochberg adjusted p-values (p.adj). Significant comparisons are indicated by an asterisk (*). C: compost; CC: cover crop; PW: pruning woods.

| **Pairwise PERMANOVA** | **F.value** | **R.squared** | **p.value** | **p.adj** |
|---|---|---|---|---|
| C vs CC | 3.0738 | 0.23511 | 0.001* | 0.003* |
| C vs PW | 1.8025 | 0.15272 | 0.091 | 0.1365 |
| CC vs PW | 0.79949 | 0.07403 | 0.623 | 0.623 |

**Supplementary Table 3**. Pairwise PERMANOVA analysis of β-diversity between experimental groups (NT and T) for samples collected in June 2022. Analyses were performed using 999 permutations. The table reports the F statistic, $R^2$ (proportion of variance explained), p-values, and Benjamini–Hochberg adjusted p-values (p.adj). Significant results are indicated by an asterisk (*).

| **Pairwise PERMANOVA** | **F.value** | **R.squared** | **p.value** | **p.adj** |
|---|---|---|---|---|
| NT vs T | 2.0148 | 0.11184 | 0.041* | 0.041* |

**Supplementary Table 4**. Pairwise PERMANOVA analysis of β-diversity between experimental groups (NT and T) for samples collected in July 2022. Analyses were performed using 999 permutations. The table reports the F statistic, $R^2$ (proportion of variance explained), p-values, and Benjamini–Hochberg adjusted p-values (p.adj). Significant results are indicated by an asterisk (*).NT: no-tillage; T: tillage.

| **Pairwise PERMANOVA** | **F.value** | **R.squared** | **p.value** | **p.adj** |
|---|---|---|---|---|
| NT vs T | 2.6097 | 0.14023 | 0.025* | 0.025* |

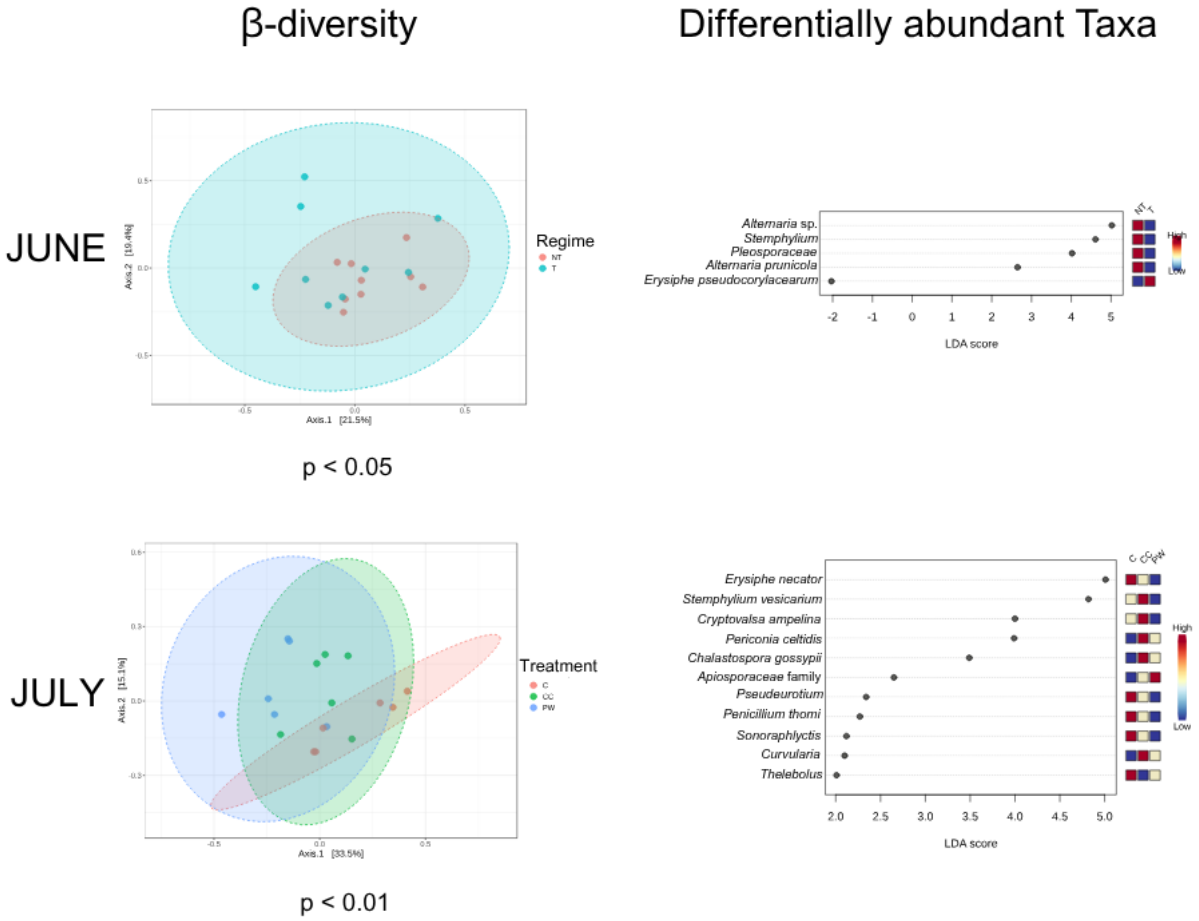


**Supplementary Figure 7.** Fungal β-diversity and differentially abundant taxa across sampling months in 2023. Principal Coordinate Analysis (PCoA) based on Bray-Curtis dissimilarity illustrates the fungal community β-diversity that was significantly different for each sampling month (May, June, and July). LEfSe analysis highlights the fungal taxa that underwent significant shifts each month in relation to the soil management practice applied: cover cropping in May and tillage in June and July.

**Supplementary Table 5**. Pairwise PERMANOVA analysis of β-diversity between experimental groups (NT and T) for samples collected in June 2023. Analyses were performed using 999 permutations. The table reports the F statistic, $R^2$ (proportion of variance explained), p-values, and Benjamini–Hochberg adjusted p-values (p.adj). Significant results are indicated by an asterisk (*). NT: no-tillage; T: tillage.

| Pairwise PERMANOVA | F.value | R.squared | p.value | p.adj |
|---|---|---|---|---|

| | | | | |
|---|---|---|---|---|
| NT vs T | 2.0034 | 0.11128 | 0.028* | 0.028* |

**Supplementary Table 6.** Pairwise PERMANOVA analysis of β-diversity among experimental groups (C, CC, and PW) for samples collected in July 2023. Analyses were performed using 999 permutations. The table reports the F statistic, $R^2$ (proportion of variance explained), p-values, and Benjamini–Hochberg adjusted p-values (p.adj). Significant results are indicated by an asterisk (*). C: compost; CC: cover crop; PW: pruning woods.

| Pairwise PERMANOVA | F.value | R.squared | p.value | p.adj |
|---|---|---|---|---|
| C vs CC | 1.6921 | 0.14472 | 0.085 | 0.085 |
| C vs PW | 4.3649 | 0.30386 | 0.009* | 0.027* |
| CC vs PW | 2.0118 | 0.16749 | 0.031* | 0.0465* |

**Supplementary Table 7.** Homogeneity of multivariate dispersions (betadisper) for fungal communities across ploughing treatments (NT = no-till, T = till) and cover crops (PW, CC, C). Average distances to group centroid (AvgDist) are shown, along with ANOVA and permutation test statistics. Significance codes: ** p < 0.01, ns = not significant.

| Cover Crop | Comparison | N° Samples | AvgDist | F_ANOVA | p_ANOVA | F_Perm | F_Perm | Significance |
|---|---|---|---|---|---|---|---|---|
| PW | NT vs T | 36 | 0.421 | 2.904 | 0.097 | 2.904 | 0.100 | ns |
| CC | NT vs T | 36 | 0.422 | 14.448 | 0.001 | 14.44 | 0.001 | ** |
| C | NT vs T | 36 | 0.466 | 3.564 | 0.068 | 3.564 | 0.066 | ns |
